# Valence-based biases in collective temporal thought: The role of question framing, culture, and age

**DOI:** 10.3758/s13421-024-01680-y

**Published:** 2025-01-14

**Authors:** Zizhan Yao, Kristi S. Multhaup, Phia S. Salter

**Affiliations:** 1 Department of Psychology, Davidson College, 209 Ridge Rd, PO Box 5000, Davidson, NC 28035 USA; 2 Psychology Faculty Services, University of California, Santa Cruz, 1156 High Street, Santa Cruz, CA 95064 USA

**Keywords:** Collective memory, Collective future thought, Aging, Positivity effect, Dialectical thinking

## Abstract

Collective temporal thought includes individuals’ memories of group experiences and expectations about the group’s collective future. Prior studies have found inconsistent valence biases (e.g., positivity) in North American collective memory and consistently negative biases in collective future thought. Discrepancies in collective memory valence biases may be due to different question framing across studies. Moreover, a limited number of studies extend collective temporal thought research beyond Western nations and few studies examine potential age-related differences in this area. Therefore, the present study investigates valence-based biases in collective temporal thought from the perspective of question framing, culture, and participant age. Participants (*N* = 1,548) included younger (20–39 years) and older (60+ years) adults from the USA and mainland China. Whereas Americans’ collective memory biases varied across question framings, Chinese participants consistently displayed positivity biases. The American bias patterns were specific to collective memory and did not carry over to collective future thought ratings. Chinese participants showed higher dialectical thinking than American participants and dialectical thinking positively correlated with the proportion of positive events reported. Older adults generated significantly more positive events than younger adults, more so in collective memory than in collective future thought. Overall, collective temporal thinking is influenced by question framing, cultural context, and participant age.

**Supplementary information:**

The online version contains supplementary material available at 10.3758/s13421-024-01680-y.

## Introduction

Collective memory and collective future thought together form collective temporal thought (Yamashiro & Roediger, 2019). This concept can be biased by various factors, such as group memberships and national propaganda, influencing how events are perceived as positive or negative. The present study focuses on valence-based biases in collective temporal thought, driven by inconsistent findings in North American contexts. We explore whether question framing contributes to these biases, address the Western-centric focus in prior research by including Chinese participants, and address the youth-centered focus in prior studies by including older as well as younger adults from both countries. In short, the present study examines collective temporal thought across three dimensions: (1) testing the impact of question framing on valence-based biases in American collective memory, using three question framings in prior research; (2) recruiting comparable American and mainland Chinese samples to explore cross-cultural differences and dialectical thinking; and (3) extending previous research by including older as well as younger adults to investigate the role of aging in collective temporal thought.

### Valence-based biases in Western collective temporal thought

Psychological research often explores collective temporal thought through event-fluency tasks, where participants list important historical events (collective memory) or imagine future events at a collective level (conceived by MacLeod et al., 1997; also used by Shrikanth et al., 2018; Shrikanth & Szpunar, 2021). To determine event valence, participants either respond to pre-assigned valence prompts (e.g., things that they were excited/worried about; Shrikanth et al., 2018) or later rate the valence of self-generated events (e.g., Shrikanth & Szpunar, 2021; Yamashiro & Roediger, 2019). Despite using similar methods, prior Western studies found mixed valence-based biases in collective memory but largely consistent biases in collective future thought (see Table 1). We consider each in turn, starting with future thought.

**Table 1:** Methods and findings of previous studies on Western collective temporal thought

Study	Participants	Question Framing	Bias in CM	Bias in CFT
Shrikanth et al. (2018)	Americans Canadians	Things they were excited/worried about for their nation’s future in the next week, year, and 5–10 years	Not explored	**Negativity bias**
Yamashiro and Roediger (2019)	Americans	Five events that brought about America as a nation + 10 events that all Americans should remember about their history + all the events in their country’s future that they are worried/excited about.	Pronounced **positivity bias** for origin events & less extreme positivity bias for normative events	**Negativity bias**
Cyr and Hirst’s (2019) Study 1	Americans British Indians	“Decide which events are expected to take place for a typical nation…imagine a hypothetical nation with a hypothetical history”	**Positivity bias** for the hypothetical nation’s CM	Not explored
Topcu and Hirst (2020)	Americans	List 15 (Study 1) + 10 (Study 2) events involving the U.S. in the past 1–50 years and 15 (Study 1) + 10 (Study 2) events in the USA’s 1–50 years of future	**Negativity biases**	No bias (*Neutral*)
Choi et al.’s (2021) Study 1	Americans Germans	10 events that they are proud of and 10 events that they are ashamed of from their own history	As many shamed events as prideful ones (*Neutral*)	Not explored
Shrikanth and Szpunar’s (2021) Studies 1 & 2	Americans	Positive and negative events that had occurred in their nation in the last week, year, 5–10 years, and ever in the past (added in Study 2)	**Negativity biases**	Not explored
Shrikanth and Szpunar’s (2021) Studies 3–4	Americans	Study 3: List as many events as they could from the public past of their nation. Study 4: List the five most important events in American history that had occurred at least more than 20 years into the past	**Negativity biases**	Not explored
Shrikanth & Szpunar’s (2021) Study 5	Americans	List positive and negative events that had happened in their nation within the last week, year, and *10 or more years ago* and list positive and negative things that could happen in their nation in the next week, year, and 10 or more years	**Negativity bias**	**Negativity bias**
Ionescu et al. (2022)	French	Adapted Yamashiro and Roediger (2019) by replacing America with France	Pronounced **positivity bias** for origin events & slight positivity bias for normative events	**Negativity bias**

*Note*. CM = collective memory; CFT = collective future thought. The table only presents studies that are relevant to the current study. Bias in CM means the valence-based biases found in collective memory by prior studies. Bias in CFT indicates valence-based biases found in collective future thought. Negativity biases are bolded, positivity biases are underlined and bolded, and neutral biases are italicized. Some studies’ designs and results are only partially exhibited in the table. Please refer to the original articles for comprehensive information about the reported studies

Shrikanth et al. (2018) were the first to compare collective futures with personal futures. In five experiments, North American participants listed things they were excited or worried about for their personal and national futures. Their results consistently showed a *negativity bias* in collective future thought, with more negative than positive events for their country’s future. This finding of negativity bias in collective future thoughts has been replicated with North American and European participants (e.g., Ionescu et al., 2022; Shrikanth & Szpunar, 2021; Yamashiro & Roediger, 2019; for an exception, see Topcu & Hirst, 2020).

Unlike the largely consistent negativity biases in Western collective future thought, valence-based biases in collective memory show discrepant results. Yamashiro and Roediger (2019) surveyed over 2,000 American participants on events that brought about America as a country (origin events) and events that they believed every American should remember (normative events) along with their personal and collective futures (see Table 1 for exact framing). Participants categorized these events as either positive or negative. They found a negativity bias in collective future thought but pronounced positivity biases for the origin and normative events. This suggests Americans view their nation's trajectory as declining, with a positive origin and a pessimistic future outlook. Ionescu et al. (2022) replicated Yamashiro and Roediger’s (2019) finding of positivity biases in Western collective memory using a French sample, but these positivity biases have not been replicated consistently in US samples. For instance, Topcu and Hirst (2020) found a negativity bias in American collective memory. Similarly, Shrikanth and Szpunar (2021) found negativity biases in five studies. They argued that using two valence scales better captured the mixed emotions of public events, but a negativity bias persisted, suggesting other factors may be at play. Given the inconsistent findings reported, the present study explores whether the exact phrasing of the prompt, which we will call *question framing*, explains these discrepancies (see Table 1).

Yamashiro and Roediger (2019) argued that origin events, which only they and Ionescu et al. (2022) prompted from participants, may be inherently more positive than other public events because they reflect a nation’s founding value and promote group cohesion. Participants may feel a moral obligation to remember those events favorably (Churchill et al., 2019). Additionally, like mostly positive life-script events, national-script events influence valence in collective thinking (Liu & Szpunar, 2023). Cyr and Hirst’s (2019) first experiment supports this interpretation. Participants from America, Britain, and India imagined events for a fictional nation’s past to its future and then rated these events on a negative-to-positive scale. Despite most events being related to wars and politics, the overall valence rating was positive. This suggests that, unlike other public events, origin events likely tap into a collective memory reservoir that fosters national identity and positive sentiments, contributing to the discrepancy in Western collective memory with a positivity bias for origin event prompts and a negativity bias for other prompts.

In summary, as shown in Table 1, most studies show a negativity bias in Western collective future thought, but mixed findings in their collective memories. Question framing may contribute to these discrepancies, which this study aimed to test. Additionally, this research investigates collective temporal thought in non-Western participants.

### Non-Western collective temporal thought

Liu and Szpunar (2023) emphasized expanding research beyond WEIRD (i.e., Western, Educated, Industrialized, Rich, and Democratic) countries (Henrich et al., 2010) to explore cross-cultural differences in collective temporal thought. Over a decade of research has shown great heterogeneity across cultures. For example, Liu et al. (2005, 2009) identified distinct differences between Eastern, particularly Chinese, and Western historical perspectives. For instance, Mao Zedong was regarded as a central figure in all six Asian samples (e.g., Japanese, Taiwanese, Malaysians) but was absent in Western samples (Liu et al., 2005). Furthermore, China was one of the only two nations among a sample of 12 countries for which the top ten public events were primarily positive. Research on non-Western countries shows a pattern of valence-based biases distinct from the Western samples’ pattern. Choi et al. (2023) found that Chinese and Malaysian participants listed predominantly positive historical events, while Western participants listed mainly negative events. Yue et al. (2023) further confirmed a positivity bias in Chinese collective past, present, and future. This valence pattern extends to collective future thinking. Deng et al. (2022) and Mert et al. (2022) found neutral and positivity biases in Chinese participants’ imagined events, compared with negativity biases in American and Turkish participants. Mert et al. recognized that social factors like China’s early success in controlling COVID-19, rising Chinese nationalism, and surging economic growth in the 21st century, may contribute to a more optimistic outlook among Chinese participants. Likewise, Hacıbektaşoğlu et al. (2023) demonstrated the negativity in Turkish collective temporal thought was influenced by temporal horizon, socio-political leanings, and culture.

The present study contributes to the effort to understand collective temporal thought beyond Western contexts and does so with the addition of including older and younger adults in two distinct cultures, as well as investigating the role of dialectical thinking in valence-based biases.

### Underlying factors impacting valence-based biases: Socio-cultural and age

#### Socio-cultural factors

Researchers have identified several socio-cultural factors that might explain the different patterns of valence-based biases they observed in Chinese participants. For example, Deng et al. (2022) suggested that the lack of bias in Chinese participants’ collective future views, contrasted with American participants’ negativity biases, may be due to cultural differences in dialectical thinking, or naïve dialecticism. Peng and Nisbett (1999) defined *naïve dialecticism* as the East Asian tendency to tolerate contradictions and expect changes in everyday life. Cross-cultural studies have shown that East Asians exhibit significantly more dialectical thinking than Caucasians. Specifically, East Asians tend to be more tolerant of change, show ambivalence and moderation in responses, and display higher emotional complexity, which is the co-occurrence of positive and negative affect (Hamamura et al., 2008; Spencer-Rodgers et al., 2004, 2009, 2010a, 2010b). Given public events are often associated with mixed emotions (Shrikanth & Szpunar, 2021), those with higher levels of dialectical thinking may exhibit distinct valence-based biases when appraising their country’s past and future (Deng et al., 2022). These cultural differences in naïve dialecticism necessitate cross-cultural studies of collective temporal thought that include measures of naïve dialecticism. The present study does so.

#### Age-related factors

Participant age can significantly influence the valence of information retrieved from the past. As people age, they often favor positive over negative information in attention and memory, known as the *positivity effect* (for a review, see Mather & Carstensen, 2005). While this effect is well documented in personal memory (e.g., Chung & Lin, 2012; Wang et al., 2015), it is less explored in collective memory. Shrikanth and Szpunar (2021) reported a significant temporal domain (memory, future) by valence interaction across all age groups (i.e., 20–29, 30–39, 40–49, 50–59, and 60+ years), all of which showed a significant negativity bias in American collective memory. However, the small sample sizes prevented analysis of bias variations by age group (they stated a minimum of eight participants in each group, p. 185).

The positivity effect has been observed in East Asian populations (e.g., Gong & Fung, 2020; Wang et al., 2020), but there are differences in age-related memory patterns between Westerners and East Asians. Wang et al. (2012) discovered that Western participants recalled public events most vividly from ages 10–19 years, while Chinese participants peaked from ages 30–39 years. Tekcan et al. (2017) also explored the presence of a “bump” in Turkish participants’ retrieval of public events, and their results suggest that a reminiscence bump may only emerge for highly important public events that partially construe a society’s collective memories. Wang (2008) emphasized understanding the age characteristics of collective memory in different cultural contexts. The present study uses an extreme age group design to assess the positivity effect in younger and older adults from various cultures and to determine if the positivity effect varies across cultures.

### Purposes of the present study

This study aimed to replicate previous findings on valence-based biases in American collective temporal thought by manipulating question framing. It also extends prior research by recruiting younger (20–39 years) and older (60+ years) American and Chinese samples to explore collective temporal thought from the perspective of age and culture. Using event fluency and future fluency tasks, the study examines the role of dialectical thinking in collective temporal thought with the Brief Dialectical Self Scale (B-DSS; Spencer-Rodgers et al., 2010a, 2010b), as suggested by Deng et al. (2022). Hypotheses include (a) a significant Temporal Domain × Framing × Country interaction in proportion positive events reported, driven by a Framing × Country interaction in collective memory that will not be present in collective future thought; (b) the Framing × Country interaction in collective memory will show Chinese participants reporting more positive events than Americans for origin and normative framings, but not for general conditions influenced by COVID-19’s recency (data collected in 2022); (c) Chinese will report a higher proportion of positive events than Americans (a main effect of country); and (d) older adults will be more positive than younger adults (a main effect of age). Based on prior studies (Peng & Nisbett, 1999; Spencer-Rodgers et al., 2004; Spencer-Rodgers et al., 2010a, 2010b), hypotheses about dialectical thinking include (a) Chinese participants will display stronger dialectical thinking than Americans and (b) dialectical thinking will be positively correlated with participants’ proportion of neutral events reported due to previously reported heightened level of emotional complexity (Spencer-Rodgers et al., 2010a, 2010b).

## Method

### Participants

The Davidson College HSIRB approved data collection in both the USA and mainland China. We hired Qualtrics (Qualtrics, Provo, UT, USA) to complete the recruiting process for both samples. The American data were collected from June to July 2022. The Chinese data were collected from August to September 2022, which strategically preceded the most significant political event of that year: the 20th National Congress of the Chinese Communist Party from 16 to 22 October 2022.

#### American sample

We conducted an a priori power analysis using G*Power version 3.1.9.7 (Faul et al., 2009) for US sample size estimation, based on data from Shrikanth and Szpunar’s (2021) Experiment 4 (*N* = 50), which compared the number of positive public events to negative public events generated by participants. The effect size in Shrikanth and Szpunar’s study was *d* = .35, considered to be small using Cohen’s criteria (Cohen, 1977). With a significance criterion of α = .05 and power = .95, the minimum sample size needed with this effect size is *N* = 688 for a mixed-factorial analysis of variance (ANOVA). Given that Spencer-Rodgers et al. (2004) reported four ethnic groups differed in their levels of dialectical thinking, the present study aimed for similar numbers of participants in each ethnic group and age cohort to investigate the role dialecticism may play in American collective temporal thought. To be included in the American sample, participants had to be aged between 20 and 39 years or 60+ years; be American residents; identify as African American, Asian American, European American, or Latino American; and pass two attention checks. Data were screened for unusable responses (e.g., gibberish, not in English) and Qualtrics replaced these participants to reach the original recruitment goals of 400 younger and 400 older adults, with 100 respondents from each ethnic group.

The final sample size was 862 (67% female) participants. There were 420 younger adults (i.e., 20–39 years) and 442 older adults (i.e., 60+ years). Detailed demographic information appears in Table 2. Most American participants (65%) received some college education (36% received some college education with no degree and 29% earned a bachelor’s degree).

**Table 2:** Demographic information of participants split by country, age, and ethnicity

Country	Ethnicity	Age group, y	n	% Female	% < High school	% High school	% Some college	% Bachelor’s degree	% Graduate degree
		20–39	348	62% (217)	1% (2^a^)	3% (11)	9% (33)	65% (225)	22% (76)
China	N/A	60+	338	45% (153)	2% (7)	29% (99)	36% (122)	31% (104)	2% (6)
		Total	686	54% (370)	1% (10)	16% (110)	23% (155)	48% (329)	12% (82)
	Asian American	20–39	110	69% (76)	2% (1^b^)	13% (14)	29% (32)	43% (47)	13% (14)
		60+	100	44% (44)	0% (0)	5% (5)	24% (24)	41% (41)	30% (30)
	African American	20–39	98	69% (68)	4% (4)	26% (25)	37% (36)	23% (23)	10% (10)
		60+	110	78% (86)	1% (0^a^)	10% (11)	39% (43)	34% (37)	16% (18)
USA	Latino American	20–39	98	76% (74)	2% (1^a^)	30% (29)	43% (42)	16% (16)	9% (9)
		60+	107	58% (62)	2% (1^a^)	21% (22)	43% (46)	21% (23)	13% (14)
	European American	20–39	114	77% (88)	3% (2^a^)	25% (29)	42% (48)	23% (26)	7% (8)
		60+	125	63% (79)	0% (0)	18% (22)	29% (36)	30% (38)	23% (29)
	Combined	20–39	420	73% (306)	2% (12)	23% (97)	38% (158)	27% (112)	10% (41)
		60+	442	61% (271)	< 1% (3)	14% (60)	34% (149)	31% (139)	21% (91)
		Total	862	67% (577)	1% (15)	18% (157)	36% (307)	29% (251)	15% (132)

*Note.* This table presents the number of participants in Chinese and American samples split by age group and ethnicity. “*n*” denotes the number of participants in each group. Education levels were partially aggregated. The category of Some College includes both some colleges with no degree and Associate’s degree. The Graduate Degree category encompasses Master’s degree, doctoral degree, and professional degree. Numbers in parentheses (e.g., 76) denote the actual number of people in the given group. Superscripts next to a number (e.g., X^a^, X^b^) indicate the number of participants who refused to identify their education level (^a^ = 1, ^b^ = 2)

#### Chinese sample

The Chinese sample included participants fluent in written and spoken Chinese, residing in mainland China, and identifying as mainland Chinese. The ethnicity distribution of the Chinese sample was not considered due to the 56 ethnicities in mainland China. To match the American sample within budget limits, we aimed for at least 300 Chinese participants per age group. Data were screened for invalid responses, and Qualtrics re-fielded when necessary.

The final sample was 686 (54% female) participants; 348 participants were younger adults, and 338 participants were older adults (see Table 2). Most Chinese participants (71%) received at least some college education (23% of Chinese participants received some college education with no degree and 48% earned a bachelor’s degree).

### Surveys

For American participants, three question-framing versions (origin framing, normative framing, general framing) of the survey were used to examine the role of framing in eliciting different biases in collective memory (see Appendix A). Thus, question framing was only manipulated in the collective memory section. While instructions did not vary across conditions in the collective future section, the groups did, in fact, start the study with different framing for remembering their group’s history. All survey versions had the same structure, which included four parts: a collective memory section, a collective future thought section, a B-DSS, and demographic questions (i.e., age, gender, ethnicity, education, and belief in American exceptionalism).

The origin and normative framings conceptually replicated the design of Yamashiro and Roediger’s (2019) probe for collective memory. The general condition conceptually replicated Shrikanth and Szpunar’s (2021), including questions about 1 year and 10 or more years in the past or future. The B-DSS, adapted from Spencer-Rodgers et al. (2010a, 2010b), contains 14 statements that describe participants’ thoughts, feelings, and behaviors, rated on a 7-point Likert scale (1 = *strongly disagree*, 7 = *strongly agree*). Cronbach’s alphas were .73 (Chinese) and .86 (European Americans) (Spencer-Rodgers et al., 2010a, 2010b). American participants’ belief in American exceptionalism was measured by the Yes/No answer to “Is America exceptional among nations” from Yamashiro and Roediger (2019).

The same surveys were used for Chinese participants with the following differences: (a) the surveys were translated into Chinese using back-translation for validity (Brislin, 1970); (b) probes asked for collective memory and future thought for China; (c) the Chinese-translated version of B-DSS was used (Shang et al., 2022); and (d) no ethnicity or American exceptionalism questions were included.

### Procedure

Participants were randomly assigned to one of the three question framings of the survey. They had 4 min to recall historical events that happened in their country based on varied framing prompts (see Appendix A for details). In the origin framing, participants listed events that shaped America/China as a nation. In the normative framing, they listed public events every American/Chinese should remember (Yamashiro & Roediger, 2019). To ensure consistency with prior research, participants in the general framing had 2 min to list events from the past year and 2 min for events from 10 or more years ago (Deng et al., 2022; Mert et al., 2022; Shrikanth & Szpunar, 2021). Regardless of framing, participants then rated each event they listed using 5-point Likert scales for positive (1 = *not very positive*, 5 = *very positive*) and negative (1 = *not very negative*, 5 = *very negative*) valences. This dual-valence scale, adapted from Shrikanth and Szpunar (2021), provided a more nuanced measure of participants’ emotional responses to public events than a single rating scale. Additionally, it allowed for the examination of emotional ambivalence toward generated events, consistent with naïve dialecticism research (e.g., Spencer-Rodgers et al., 2010a, 2010b).

The collective future thought section required participants to report events that they imagined would happen in the nation’s future within 4 min. No framing was manipulated in the collective future thought section, although the groups did differ in how they were instructed to think about collective memory immediately prior to this section. As in the collective memory section, the general framing included two timeframes (1 year vs. 10+ years) for consistency with prior literature. All participants rated the events they generated using the dual valence scale. Lastly, they answered the B-DSS and demographic questions (both untimed).

### Data cleaning

Data cleaning was necessary to exclude invalid responses (e.g., “peace,” “native Americans”) before data analysis. Development of the coding scheme used pilot data from both Chinese and American participants. Coders excluded participants’ responses that were gibberish, vague, incomplete, incoherent, or happened outside the target country. In addition, coders carefully reviewed each event in the general framings to ensure it was appropriately categorized based on the specified timeframe and removed those that happened outside the specified timeframe. Items valid across both timeframes (e.g., climate change could reasonably be expected to occur within both 1 year and 10 years in the future) were retained if they were contextually appropriate for each timeframe.

To ensure coding reliability, the first author recruited six independent coders (three for American data and three for Chinese data) who were unaware of the hypotheses and conditions. Each coder was assigned to one condition (based on language proficiency in English and Chinese) and used OpenRefine, an open-source contextual data analysis software, to sort invalid responses based on the coding scheme (see https://openrefine.org/). The first author randomly chose 10% of data from each condition of the Chinese and American data sets for training. Interrater agreements measured by Cohen’s *kappa* (*κ*) were calculated and compared to agreed standards (Ranganathan et al., 2017). All three conditions reached moderate to substantial agreement (American origin: *κ* = 0.72, American normative: *κ* = 0.74, American general: *κ* = 0.80; Chinese origin: *κ* = 0.55, Chinese normative: *κ* = 0.82, Chinese general: *κ* = 0.63), so training was considered complete. Disagreements were resolved through discussions (a third coder was used to break the tie if necessary).

The first author updated and clarified the coding scheme according to the coders’ feedback. A new 10% of data were coded. Interrater agreements significantly improved with all conditions at or around near-perfect agreements (American origin: *κ* = 0.91, American normative: *κ* = 0.91, American general: *κ* = 0.92; Chinese origin: *κ* = 0.74, Chinese normative: *κ* = 0.82, Chinese general: *κ* = 0.89). Mutually identified invalid responses along with corresponding valence ratings were excluded from further analysis. Given the high agreement rates, all independent coders stopped coding when they finished 20% of the data from each condition and the rest of the data-coding process was completed by the first author based on the updated coding scheme.

### Data scoring

To determine valence ratings for each condition’s collective memories and future thoughts, each discrete event’s valence scores were calculated by subtracting the negative valence rating from the positive valence rating (Shrikanth & Szpunar, 2021). Events with a positive difference score (i.e., above 0) were categorized as positive, events with a negative difference score (i.e., below 0) were categorized as negative, and events with a difference score of 0 were categorized as neutral valence. Instead of excluding events that were neutral from analysis as Shrikanth and Szpunar (2021) did, these events were also analyzed along with other events to assess potential cultural differences in displaying emotional ambivalence toward past and future events.

To compare valence-based biases across temporal domains, the scoring measures from Yamashiro and Roediger (2019) were used. They operationalized each valence-based bias as the average proportion of positive events out of all reported events for each (e.g., origin collective memory, normative collective future): Mean Proportion Positive = Count_positive_/(Count_positive_ + Count_negative_+ Count_neutral_). A ratio above 0.5 indicates the presence of a positivity bias whereas a ratio below 0.5 indicates the presence of a negativity bias. A ratio of 0.5 indicates the absence of any valence-based bias (i.e., neutral).

To calculate participants’ levels of naïve dialecticism as assessed by the B-DSS, the scoring of raw points was adapted from Spencer-Rodgers et al. (2004). Since the B-DSS includes reversed statements (e.g., *I am the same around my family as I am around my friends*) in addition to non-reversed ones, participants’ scores on non-reversed statements and on reversed statements were calculated differently: reversed item score = 8 – raw score, non-reversed item score = raw score. For the overall B-DSS scale score, participants’ scores were added up and divided by 14. The higher their score was, the higher the participant’s dialectical thinking level.

## Results

### Mean proportion positive events

**Fig. 1:**
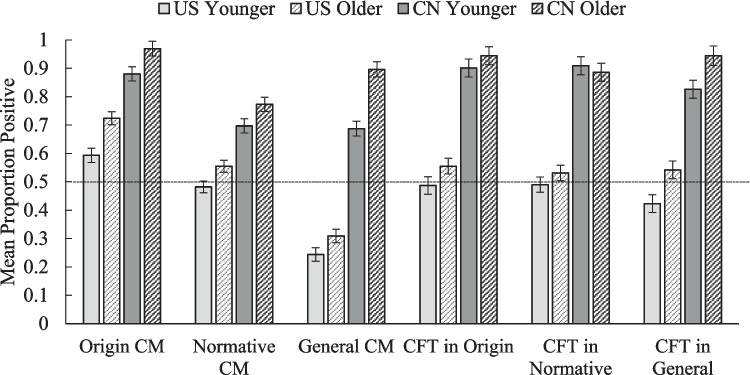
Valence-based biases for each country’s age groups. Origin CM denotes collective memory in the origin question framing; Normative CM means collective memory in the normative question framing; General CM indicates collective memory in the general question framing. Since collective future thought probes were not framed, CFT in Origin stands for the collective future thought probe used along with the origin collective memory probes. Error bars represent standard errors. The horizontal line represents the reference line of 0.5. Bars above the reference line show positivity biases; bars below indicate negativity biases. Error bars represent the standard error of the mean

As planned, a 2 (temporal domain: collective memory or collective future thought) × 3 (question framing: origin, normative, general) × 2 (age: younger adults or older adults) × 2 (country: USA or China) omnibus analysis of variance (ANOVA) with temporal domain as a within-participants variable was conducted on proportion positive events reported (see Fig. 1). Post hoc comparisons for significant effects were performed with the Least Significant Difference (LSD) test. Relevant 95% confidence intervals for mean differences are reported. All effects related to question framing and aging are discussed below. In addition, there was a significant main effect for temporal domain, *F*(1, 1532) = 27.76, *p* < .001, *η*^*2*^_*p*_ = .018, 95% CI [.03, .07], reflecting that participants gave more positive ratings for future events (*M* = .68, *SD* = .40) than for past events (*M* = .64, *SD* = .35).

### Question-framing effect in collective memory

The first aim was to assess whether question framing affected ratings. In general, it did, *F*(2, 1532) = 45.32, *p* < .001, *η*^*2*^_*p*_ = .056. Participants had the highest proportion of positive events in the origin framing (*M* = .73, *SD* = .29), lower proportion in the normative framing (*M* = .64, *SD* = .30), and the lowest in the general framing (*M* = .59, *SD* = .34). All pairwise comparisons were significant (*p* < .001), and the confidence intervals for mean differences between the origin framing and the general framing, the origin framing and the normative framing, and the normative framing and the general framing were 95% CI [.12, .19], 95% CI [.06, .12], and 95% CI [.03, .09], respectively. This main effect was qualified by two two-way interactions (Question Framing × Temporal Domain, *F*(2, 1532) = 40.96, *p* < .001, *η*^*2*^_*p*_ = .051; and Question Framing × Country, *F*(2, 1532) = 14.12, *p* < .001, *η*^*2*^_*p*_ = .018) that, along with an additional two-way interaction (Temporal Domain × Country, *F*(1, 1532) = 10.65, *p* < .001, *η*^*2*^_*p*_ = .007) were qualified by the three-way interaction, Question Framing × Country × Temporal Domain, *F*(2, 1532) = 19.35, *p* < .001, *η*^*2*^_*p*_ = .025. As expected, this three-way interaction was driven by a Question Framing × Country interaction in collective memory only, *F*(2, 1536) = 41.69, *p* < .001, *η*^*2*^_*p*_ = .051, and no such interaction in collective future thought, *F*(2, 1532) = .08, *p* = .920. The interaction is driven by the general framing being different from the other two conditions, as predicted. Contrary to the prediction, however, the general framing showed a *greater* (predicted lesser) country difference than the other two conditions (see below). Overall, these differences demonstrated a question framing effect only in collective memory since question framing was not manipulated in the collective future thinking, confirming the hypothesis that question framing contributes to prior valence discrepancies in collective memory across published studies.

### Chinese versus American collective temporal thought

**Fig. 2:**
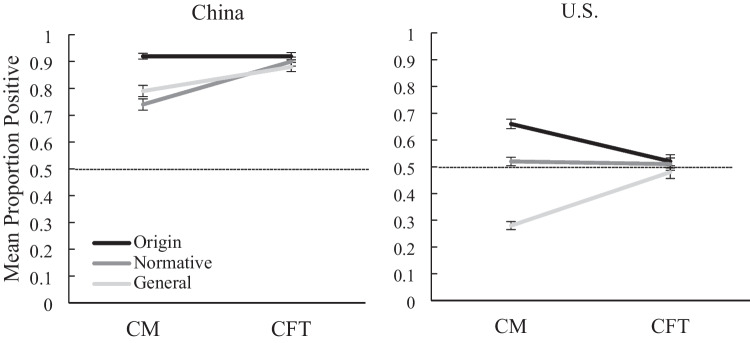
Valence-based biases in Chinese and American collective temporal thought. Chinese data are presented on the left and American on the right. Origin means the origin question framing. CM means collective memory and CFT means collective future thought. Normative denotes the normative framing. General indicates the general framing. The horizontal line represents the reference line of 0.5. Points above the reference line show positivity biases; points below indicate negativity biases. Error bars represent the standard error of the mean

The second aim of this study was to understand whether Chinese and American collective temporal thought differed in terms of valence-based biases. As expected, participants in China (*M* = .86, *SD* = .23) made more positive ratings than participants in the USA (*M* = .50, *SD* = .28), *F*(1, 1532) = 841.42, *p* < .001, *η*^*2*^_*p*_ = .355, 95% CI [.34, .39]. Following up with the significant Question Framing × Country interaction in collective memory (reported above), pairwise comparisons showed that Chinese participants (CN) generated a significantly higher proportion of positive memory events than Americans (US), with the size of this difference varying across the origin (CN: *M* = .92, *SD* = .17; US: *M* = .66, *SD* = .30; 95% CI [.19, .33]), normative (CN: *M* = .74, *SD* = .32; US: *M* = .52, *SD* = .30; 95% CI [.15, .28]), and general framings (CN: *M* = .79, *SD* = .31; US: *M* = .28, *SD* = .23; 95% CI [.44, .58]). Furthermore, separate one-sample *t*-tests (using the .5 ratio as the test value) for each Chinese (Fig. 2 left side) and US condition (Fig. 2 right side) were conducted to detect the presence of significant valence-based biases in collective temporal thought. Chinese in the origin framing displayed significant positivity biases in collective memory (*M* = .92, *SD* = .17), *t*(232) = 37.40, *p* < .001, 95% CI [.40, .45], *d* = 2.45, and in collective future thought (*M* = .92, *SD* = .22), *t*(231) = 29.50, *p* < .001, 95% CI [.39, .45], *d* = 1.93. Given both the past and future events’ ratings were very close to 1, the Chinese origin framing showed a ceiling effect, thus displaying a (possibly deceiving) flat trajectory. Chinese participants in the normative framing exhibited positivity biases for past events (*M* = .74, *SD* = .32), *t*(233) = 11.20, *p* < .001, 95% CI [.19, .28], *d* = .73, and for future events (*M* = .90, *SD* = .26), *t*(232) = 23.60, *p* < .001, 95% CI [.37, .43], *d* = 1.55, and so did Chinese participants in the general framing (past *M* = .79, *SD* = .31, *t*(218) = 13.60, *p* < .001, 95% CI [.25, .33], *d* = .92; future *M* = .88, *SD* = .26,* t*(218) = 22.00, *p* < .001, 95% CI [.35, .42], *d* = 1.49)]. Paired-sample *t*-tests indicated that different from the origin framing’s trajectory, the normative, *t*(232) = 7.44, *p* < .001, 95% CI [.12, .20], *d* = .49, and general framings, *t*(218) = 5.30, *p* < .001, 95% CI [.06, .13], *d* = .36, demonstrated rising representational trajectories from China’s past toward its future.

In contrast, American participants displayed three valence-based biases for past events. The *t*-tests revealed a positivity bias in the origin framing’s collective memory (*M* = .66, *SD* = .30), *t*(276) = 8.98, *p* < .001, 95% CI [.13, .20], *d* = .54, and a negativity bias in the general framing’s collective memory (*M* = .28, *SD* = .23), *t*(249) = −15.40, *p* < .001, 95% CI [.19, .25], *d* = .97. The normative framing displayed no bias in collective temporal thought (i.e., neutral). Overall, Americans’ representational trajectory was susceptible to the framing effect due to differences in collective memory. Paired-sample *t*-tests showed that the normative framing showed a flat trajectory, whereas the general, *t*(249) = 8.23, *p* < .001, 95% CI [.16, .26], *d* = .52, and origin framing, *t*(275) = −4.84, *p* < .001, 95% CI [.08, .20], *d* = .29, presented trajectories with opposite directions, increasingly positive and increasingly negative, respectively.

Compared with prior studies, the present study replicated both the declining trajectory of representation found in Yamashiro and Roediger (2019), who used origin framing, and the negativity bias in American collective memory discovered by prior studies, which used general framing (e.g., Choi et al., 2021; Shrikanth & Szpunar, 2021). However, we did not find a slight positivity bias in American collective memory as Yamashiro and Roediger did when using normative framing. The lack of valence-based biases in American collective future thought differed from previous findings of negativity biases (Shrikanth et al., 2018; Shrikanth & Szpunar, 2021), but replicated Topcu and Hirst’s (2020) results of neutral ratings.

### Age-related differences in collective temporal thought

**Fig. 3:**
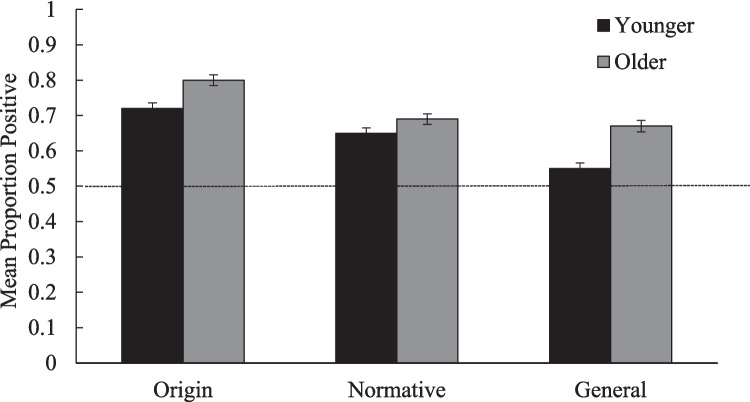
Proportion of positive events across Age × Question-framing groups. Proportion of positive events reported by younger and older participants in each question framing. Younger means the younger participants. Older denotes the older participants. The horizontal line represents the reference line of 0.5. Points above the reference line show positivity biases; points below indicate negativity biases. Error bars represent the standard error of the mean

**Fig. 4:**
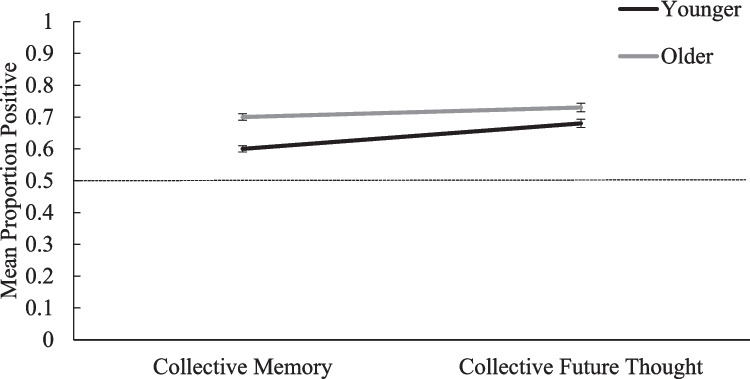
Proportion of positive events reported by younger and older participants for collective memory and collective future thought. Younger means the younger participants. Older denotes the older participants. The horizontal line represents the reference line of 0.5. Points above the reference line show positivity biases; points below indicate negativity biases. Error bars represent the standard error of the mean

The third aim was to assess whether there were age-related patterns in participants’ collective temporal thought. As predicted, older participants gave more positive ratings (*M* = .70, *SD* = .31) than younger participants (*M* = .62, *SD* = .31), *F*(1, 1532) = 44.55, *p* < .001, *η*^*2*^_*p*_ = .028, 95% CI [.06, .11]. There were two interactions involving age. First, the ANOVA revealed a significant Question Framing × Age interaction, *F*(2, 1532) = 3.93, *p* = .020, *η*^*2*^_*p*_ = .005. As shown in Fig. 3, pairwise comparisons indicated that the positivity effect (i.e., older people making more positive ratings than younger people) was more pronounced in the general (older *M* = .65, *SD* = .34; younger *M* = .54, *SD* = .32; 95% CI [.08, .17]) and origin (older *M* = .78, *SD* = .28; younger *M* = .71, *SD* = .29; 95% CI [.04, .13]) framings, both age comparisons *p* < .001, than in the normative framing (older *M* = .66, *SD* = .31; younger *M* = .62, *SD* = .29; 95% CI [.00, .08]), *p* = .048. In other words, while the size of the age-related positivity effect varies across framings, it was significant in all three framings. Furthermore, the Temporal Domain × Age interaction was significant as well, *F*(1, 1532) = 5.29, *p* = .022, *η*^*2*^_*p*_ = .003. As shown in Fig. 4, the age-related positivity effect was stronger in collective memory (older *M* = .69, *SD* = .34; younger *M* = .59, *SD* = .34; 95% CI [.08, .13]) than in collective future thought (older *M* = .71, *SD* = .39; younger *M* = .66, *SD* = .40; 95% CI [.03, .10]), but significant in both temporal domains (*ps* < .003).

### Naïve dialecticism and collective temporal thought

An independent-sample *t*-test showed that there was a significant difference between Chinese and American participants’ dialectical thinking levels, *t*(1546) = −19.79, *p* < .001, *d* = .78). Chinese participants (*M* = 3.85, *SD* = .66) had significantly higher DSS scores than American participants (*M* = 3.06, *SD* = .87), thus supporting the hypothesis that Chinese participants would display stronger dialectical thinking than Americans. The distribution of DSS scores split by country is reported in the Online Supplemental Material.

Pearson correlations were first conducted between all participants’ (both American and Chinese combined) DSS scores, and the proportion of positive, negative, and neutral events reported by them. We hypothesized a positive correlation between DSS scores and the proportion of neutral events reported, based on the notion of naïve dialecticism. Contrary to the prediction, the results showed that DSS was positively correlated with the proportion of positive events reported in collective temporal thought (memory *r* = .20, *p* < .001; future *r* = .19, *p* < .001), negatively correlated with the proportion of negative events reported (memory *r* = -.19, *p* < .001; future *r* = -.20, *p* < .001), and not correlated with the percentage of neutral events reported (memory *r* = -.05, *p* = .071; future *r* = .01, *p* = .729). Thus, the higher the level of dialectical thinking participants had, the more positive events and the fewer negative events they reported for collective temporal thought.

Separate Pearson correlations were also conducted for Chinese and American data. Similar to the analyses for the full sample, the correlations for each country separately did not support the prediction of a positive correlation between DSS scores and the proportion of neutral events reported. In fact, Americans exhibited no significant correlations in either temporal domain. The pattern for Chinese participants was more complex. Specifically, Chinese participants’ DSS scores were not correlated with neutral future events (*r* = .03, *p* = .376), negatively correlated with their percentage of positive events reported for the future (*r* = -.13, *p* < .001), and positively correlated with their proportion of negative events reported for the future (*r* = .13, *p* < .001). In other words, higher levels of dialectical thinking among Chinese participants were associated with generating fewer positive events and more negative events for China’s future. Despite the overall positivity biases in Chinese collective future thought discussed earlier, dialectical thinkers appear more likely to integrate negative events into their thoughts about the future. By contrast, Chinese DSS scores were not correlated with generating neutral, positive, or negative events for collective memory.

## Discussion

The contribution of the current study is threefold. First, the significant Temporal Domain × Question Framing × Country interaction that we predicted and found confirmed the role of question framing in eliciting different valence-based biases in collective memory (details regarding the pattern are outlined below). Therefore, when investigating valence-based biases in collective temporal thought, researchers should not only use consistent framing for replication purposes but also choose the framings that are appropriate for their particular research questions. Second, the current study extended prior research on cultural differences in collective temporal thought by considering cross-national differences. It revealed distinct representational trajectories for China and the USA. Third, the present study expanded scholarly understanding of the positivity effect by showing the presence of such an effect beyond the well-researched scope of personal cognition to the domain of collective temporal thought. This finding demonstrates one of the many connections that link humans’ individual memory with public memory.

### American collective temporal thought

#### The question-framing effect in the past but not in the future

 The present American data showed that the three question framings elicited distinctive valence-based biases in collective memory. We found no question-framing effects for *future* events, although that may not be surprising given that the framing instructions were only present in the collective memory section. Two framings, the origin and general, replicate past reports and thus simultaneously demonstrate that framing drives discrepant valence-based patterns. When prompted to think about the nation’s origin, participants in the USA show a positivity bias (present data; Yamashiro & Roediger, 2019). Origin stories may be unique in representing a cluster of events upon which societal charters of a nation are built (Hilton & Liu, 2017), perhaps due to a national life script (see Cyr & Hirst, 2019; Liu & Szpunar, 2023). By contrast, when prompted to recall general events that happened in the nation’s past, participants in the USA exhibit a negativity bias (present data; Shrikanth & Szpunar, 2021; Topcu & Hirst, 2020). When prompted to identify historical events that all Americans should remember using the normative framing, US participants showed no bias (neutral), which fails to replicate the slight positivity biases found in prior Western studies (Ionescu et al. 2022; Yamashiro & Roediger, 2019) but fits with Choi et al.’s (2021) neutral bias. Two factors may contribute to this discrepancy, one temporal and one methodological.

First, a changing social and political climate may result in the current neutral bias for normative events, at least in the case of American collective memory. Yamashiro and Roediger (2019) collected their data over a 2-week period in November 2017, whereas the current study’s data were collected during Summer 2022. It is possible that the emergence of negative social and political events (e.g., mass shootings and Trump-related events) coupled with the COVID-19 pandemic incorporated more negative events into people’s representation of normative events, thus causing a shift from a slight positivity bias to a neutral bias.

The other factor concerns methodological differences. The present study differs from Ionescu et al. (2022) and Yamashiro and Roediger (2019) regarding task ordering. Prior studies that reported a positivity bias in a normative framing (Ionescu et al., 2022; Yamashiro & Roediger, 2019) instructed participants first to report origin events and then normative events (i.e., question framing was manipulated within participants). The proximity of the two tasks may prompt participants to continue reporting more positive events even after moving away from the origin events (i.e., a carryover effect). The present study, however, assigned people to only one type of public event (i.e., conditions were manipulated between participants). Given that the framing effect is clearly observed in the present study, future research should consider using between-participants designs to avoid potential carryover effects.

### Chinese collective temporal thought

#### Positivity biases in the past and future

Whereas American participants showed a positivity bias only for memory in the origin framing, Chinese participants demonstrated a positivity bias in all three question framings for memory *and* future thought. In other words, the robust question-framing effect found in the American sample for collective memory was not found in the Chinese sample. Why might Chinese valence-based biases be resistant to the framing effect, particularly in collective memory? One possibility is that Chinese people hold inherently more positive collective memory in general than Westerners (Choi et al., 2023; Liu et al., 2009), they are prone to exhibit a positivity bias even for the types of events that are not as positively biased as the origin ones. Indeed, the results revealed positivity biases, albeit varying in size, across all the Chinese framings and temporal domains.

The current study’s positivity biases in Chinese collective future thought replicated Mert et al.’s (2022) finding of the Chinese anticipating a more positive future than Americans but contradicts Deng et al. (2022), who reported negativity bias in American collective future thought but no bias in a Chinese one. Methodological differences may elucidate the contradictory findings. Deng and colleagues used pre-assigned valence to let participants report events they would be excited and worried about for their nation’s future. By contrast, the current study included no valence information (i.e., *excited* or *worried*) in prompts, so that participants could recall positive and negative events simultaneously and later rate them on valence scales. In addition, the current procedures resembled those of Mert et al., who also did not express valence in their questions and utilized a scale to measure event valence. Taken together, it is likely that the failure to replicate Deng et al. was due to methodological differences across studies. This reinforces our point that when seeking to replicate prior findings about collective memory, care must be taken to use the same question framing.

#### Underlying factors for cross-cultural differences

Chinese participants displayed much more positive collective temporal thought than their American counterparts. Deng et al. (2022) emphasized dialectical thinking as a potential contributing factor. To explore this assumption, the current study employed the B-DSS scale to measure Chinese and American participants’ levels of dialectical thinking. Consistent with previous studies (e.g., Peng & Nisbett, 1999; Spencer-Rodgers et al., 2004), Chinese participants had significantly stronger dialectical thinking than Americans. Empirically, dialectical thinking is associated with experiencing the co-occurrence of positive and negative emotions (Spencer-Rodgers et al., 2010a, 2010b). Correlational tests revealed that participants’ DSS scores did not correlate with their proportion of neutral events reported either when Chinese and Americans were combined or when separated, potentially due to a restricted range issue (relatively few neutral events reported). Among Chinese participants, higher levels of dialectical thinking correlated negatively with the proportion of positive events reported for China’s future, with no significant correlation observed for past events. This aligns with the concept of dialectical thinking – a tendency to tolerate contradictions and anticipate changes in daily life. As Deng et al. (2022) mentioned in their discussion, stronger dialectical thinkers are more likely to have a balanced view of the collective future and less likely to envision an overwhelmingly positive future for their country. Conversely, individuals with lower levels of dialectical thinking tend to imagine a more optimistic future, potentially aligning with the preferences of the Chinese government. Thus, the more dialectical a Chinese participant is, the greater the likelihood of imagining negative future events, as reflected in the observed negative correlation.

Aside from the potential effect of dialectical thinking, various social factors may contribute to the observed valence pattern. For example, Liu and Szpunar (2023) recognized the influence of news on shaping people’s collective temporal thought. Chinese media differ from Western media in two significant ways: the positive portrayal of state image and the state-controlled nature (Schneider, 2019). Since people often use news media to acquire access to public events, a rosy depiction of the nation will most likely link public events with positive sentiments. Concurring with this notion, Mert et al. (2022) acknowledged that China’s increasing propaganda on nationalism and its superior economic performance could lead to more positive Chinese collective future thought. Conversely, the more diverse Western news coverage along with its over-emphasis on negative events may synergistically lead to the more negative American collective temporal thought (Liu & Szpunar, 2023). Perhaps this assumption was truer during the time of COVID-19. While the pandemic imposed a profound negative impact on people’s daily experiences globally, Chinese news focus has been diverted to its early success in COVID-19 control (Mert et al., 2022). It is not surprising that Chinese people would report more positive than negative collective memories.

#### The role of the positivity effect in collective temporal thought

Previous research has often sought to explore the connections between memory and future thinking within both the personal and the collective domains (e.g., Deng et al., 2022; Shrikanth et al., 2018), but few studies have explored the relationship between aging and memory in the collective domain (Teckan et al., 2017). The present research extended prior studies of individual memory by investigating the role of the *positivity effect* in collective temporal thought (Chung & Lin, 2012; Wang et al., 2015). Overall, the results confirmed the presence of a positivity effect in collective temporal thought because older adults (ages 60+ years) rated a significantly higher portion of events as positive than younger adults (ages 20–39 years). Furthermore, age interacted with other variables. Specifically, the Question Framing × Age interaction showed that the size of the positivity effect was greater in the general and origin framings than in the normative framings – but it was significant in all three conditions. Additionally, the Temporal Domain × Age interaction revealed that although older participants were overall more positive than younger participants, the size of their positivity effect was even bigger in collective memory than in collective future thought. Given the influence of age groups on the data patterns, future research should incorporate this factor into their designs.

## Limitations and future directions

First, the use of an extreme age group design limits our discussion of understanding age-related differences in collective temporal thought because longitudinal designs are required to study age-related changes. Moreover, considering that nations actively shape national memory and narratives through top-down processes like the education system and government-controlled media, it becomes crucial to explore how collective temporal thought evolves across various life stages, spanning from childhood through adolescence to adulthood. Future longitudinal research is needed to explore possible age-related changes.

A second limitation regards using difference scores as the valence criterion in our study. This approach lacks nuance, as it does not distinguish between difference scores at both ends of the rating spectrum (e.g., difference ratings 4 and 3 are treated the same as 2 and 1). To address this in light of mixed findings with dialectical thinking, we conducted exploratory analyses where we re-evaluated the data by defining emotionally ambivalent events as those with both positive and negative scores ≥ 4, indicating strength in the co-occurrence of both positive and negative ratings of events. However, only a small number of participants (10%) had responses meeting this definition of emotional ambivalence; therefore, drawing reliable conclusions from this alternative analysis was limited. A more robust examination with targeted sampling is needed to grasp the potential relationship between dialectical thinking and generation of emotionally ambivalent temporal events.

Another potential issue is the lack of counterbalancing between the temporal domain in our study. Although we did not counterbalance the order of the CM and the CFT sections of the survey, the concern over the potential carryover effect from the retrieval of memory events to the imagining of future events can be addressed by examining the data pattern. As seen in Fig. 2, regardless of the type of collective memory framing prompts they received, participants’ valences for future events converged, indicating the absence of carryover effects (e.g., CFT following origin and normative framings in CM should be more positive than that following the general framing).

Relatedly, the current research’s inclusion of two timeframes in the general condition may introduce additional questions about how timeframes may interact with all of the different question framings. Nonetheless, because our primary aim was to compare the different approaches from prior studies (i.e., studies with general framings used different time frames, but origin framings did not; Deng et al., 2022; Mert et al., 2022; Shrikanth & Szpunar, 2021), we did not vary timeframes across all the framings. This remains an important question for future research.

Lastly, an important consideration is that the American sample in the current study included a racially diverse set of participants. Our preliminary quantitative analysis suggests that different ethnic sub-groups in the American sample may show different rating trajectories in collective temporary thought (Yao et al., in preparation). This suggests that there are still more factors to consider as researchers try to fully grasp the various dimensions of American collective temporal thought.

## Conclusion

This study identified significant interactions among framing, age, culture, and temporal domain in influencing valence-based biases in collective temporal thought. Specifically, we expected and found the question-framing effect for collective memory. The lack thereof in collective future thinking is not surprising since question framings were not manipulated again in that section of the surveys. Chinese participants consistently displayed positivity biases across all framings and temporal domains, while Americans showed varied biases depending on the question framing used. Additionally, older adults were more positive about collective temporal thought than younger adults, with this positivity effect being more pronounced in collective memory than in collective future thought.

The interaction between question framing, culture, and temporal domain highlights how different societies interpret collective memories and future thoughts through distinct lenses influenced by their historical narratives and socio-political contexts. The positivity bias among Chinese participants may reflect more favorable collective representations, though this may not be a purely cultural inclination given the unexpected, correlational dialectical thinking results. State-controlled media or other social factors, rather than cultural leanings alone, may contribute to the observed pattern. In contrast, the varied biases in American participants underscore the role of question framing in shaping collective temporal thought, revealing a susceptibility to negativity bias when recalling general historical events. Moreover, the interaction between age and temporal domain showed that older adults' positivity effect was stronger in collective memory than in collective future thought. This suggests that as people age, they may view public events more positively, which can have implications for understanding how collective memories are constructed and maintained over time.

Together, these findings offer new empirical insights into the factors that shape biases in collective temporal thought. Question framing provides an important lens for understanding when biases in collective temporal thought emerge and in which direction. This work also highlights the importance of including diverse samples (e.g., age, culture) in this growing area of research. The inclusion of diverse samples provided additional insights into the different trajectories possible in collective temporal thought.

## Supplementary information

Below is the link to the electronic supplementary material.

Supplementary file1 (DOCX 57 KB)

## Data Availability

The datasets generated and/or analyzed during the current study are not publicly available due to ongoing qualitative analysis but are available from the corresponding authors on reasonable request. The experiment was not pre-registered; however, hypotheses were generated, and analyses were planned before data collection for a thesis project.
