# Supplementary material for: Valence-based biases in collective temporal thought: The role of question framing, culture, and age
Source: Mem Cognit. 2025 Jan 14;53(6):1738–53. doi: 10.3758/s13421-024-01680-y (PMC12402011; doi:10.3758/s13421-024-01680-y)

**Appendix A**

**Origin Question Framing**

***Collective Memory Section***

What are the origins of America [China]? Please use as much of the next 4 minutes as possible to list historical events that brought about America [China] as a nation. Please make your list on your own without help from anyone or anything else. This page will not advance until the time is reached. (*Spaces provided for up to 15 events and events were individually represented for valence ratings*)

***Collective Future Thought Section***

What do you envision for the future of America [China]? Please use as much of the next 4 minutes as possible to list events that you think may happen to the nation in the future. Please make your list on your own without help from anyone or anything else. This page will not advance until the time is reached.

(*Spaces provided for up to 15 events and events were individually represented for valence ratings*)

**General Question Framing**

***Collective Memory Section***

What do you know about America [China]'s past? Please use as much of the next 2 minutes as possible to list historical events that happened to America [China] (during the last year/10 or more years ago). Please make your list on your own without help from anyone or anything else. This page will not advance until the time is reached.

(*Spaces provided for up to 15 events and events were individually represented for valence ratings*)

***Collective Future Thought Section***

What do you envision for the future of America [China]? Please use as much of the next 2 minutes as possible to list events that you think may happen to the nation (in the next ONE year/10 or more years from now). Please make your list on your own without help from anyone or anything else. This page will not advance until the time is reached.

(*Spaces provided for up to 15 events and events were individually represented for valence ratings*)

**Normative Question Framing**

***Collective Memory Section***

What historical events should all Americans [Chinese] remember about their nation? Please use as much of the next 4 minutes as possible to list historical events that you believe every American [Chinese] should remember. Please make your list on your own without help from anyone or anything else. This page will not advance until the time is reached.

(*Spaces provided for up to 15 events and events were individually represented for valence ratings*)

***Collective Future Thought Section***

What do you envision for the future of America [China]? Please use as much of the next 4 minutes as possible to list events that you think may happen to the nation in the future. Please make your list on your own without help from anyone or anything else. This page will not advance until the time is reached.

**Supplementary Data**

**Counts of Generated Events**

In total, American participants generated 6289 eligible memory events (younger: 3248, older: 3041) and 5485 future events (younger: 2541, older 2944). Chinese participants produced 3115 memory events (younger: 2087, older: 1028) and 2615 future events (younger: 1648, older: 967). See the table below for a detailed breakdown by age, question framing, and temporal domain.

|  | Origin CM | | Normative CM | | General CM | | Origin CFT | | Normative CFT | | General CFT | |
| --- | --- | --- | --- | --- | --- | --- | --- | --- | --- | --- | --- | --- |
|  | 20-39 | 60+ | 20-39 | 60+ | 20-39 | 60+ | 20-39 | 60+ | 20-39 | 60+ | 20-39 | 60+ |
| US | 878 | 949 | 1336 | 1215 | 1034 | 877 | 674 | 917 | 8922 | 1012 | 975 | 1015 |
| CN | 644 | 264 | 827 | 395 | 616 | 369 | 543 | 264 | 503 | 318 | 602 | 385 |

**Omnibus ANOVA without General Framing**

A 2 (temporal domain: collective memory or collective future thought) × 2 (framing: origin, normative) × 2 (age: younger adults or older adults) × 2 (country: US or CN) omnibus analysis of variance (ANOVA) with temporal domain as a within-participants variable was conducted on the proportion of positive events reported without the general framing. There were significant main effects of country [*F*(1, 1067) = 433.84, *p* < .761, *η^2^_p_* = .034], question framing [*F*(1, 1067) = 35.69, *p* < .001, *η^2^_p_* = .032], and age [*F*(1, 1067) = 16.56, *p* < .001, *η^2^_p_* = .015], but no main effect of temporal domain [*F*(1, 1067) = .09, *p* = .761, *η^2^_p_* = .034]. Additionally, significant interactions included: Temporal Domain × Country [*F*(1, 1067) = 37.53, *p* < .001, *η^2^_p_* = .034], Temporal Domain × Question Framing [*F*(1, 1067) = 34.58, *p* < .001, *η^2^_p_* = .031], and Temporal Domain × Age [*F*(1, 1067) = 5.66, *p* = .018, *η^2^_p_* = .005]. No significant interactions between Question Framing × Age [*F*(1, 1067) = 1.85, *p* = .174, *η^2^_p_* = .002], Question Framing × Country [*F*(1, 1067) = 1.09, *p* = .174, *η^2^_p_* = .001], and Question Framing × Country × Temporal Domain [*F*(1, 1067) = 0.70, p = .404, *η^2^_p_* = .001] were found.

**Omnibus ANOVA Using Positive Ratings Only**

Per the reviewer’s request, we re-ran the 2 (temporal domain: collective memory or collective future thought) × 3 (question framing: origin, normative, general) × 2 (age: younger adults or older adults) × 2 (country: US or CN) omnibus analysis of variance (ANOVA) with temporal domain as a within-participants variable only using the positive rating participants responded to their list of generated events, instead of using the original proportion positive measure. The overall data pattern did not change when proportion positive measures were replaced with valence ratings. Detailed *F-tests* results are shown in the table below.

| Effect | *F* | d*f* | *p* | *η^2^_p_* |
| --- | --- | --- | --- | --- |
| Temporal Domain | 37.42 | (1, 1523) | < .001 | .024 |
| Temporal Domain × Country | 20.27 | (1, 1523) | < .001 | .013 |
| Temporal Domain × Question Framing | 44.95 | (2, 1523) | < .001 | .056 |
| Temporal Domain × Age | 8.70 | (1, 1523) | .003 | .006 |
| Temporal Domain × Country × Question Framing | 22.42 | (2, 1523) | < .001 | .039 |
| Country | 885.93 | (1, 1523) | < .001 | .368 |
| Question Framing | 44.70 | (2, 1523) | < .001 | .055 |
| Age | 42.65 | (1, 1523) | < .001 | .027 |
| Country × Question Framing | 18.50 | (2, 1523) | < .001 | .024 |
| Country × Age | 4.25 | (1, 1523) | .039 | .003 |

**Timeframe Analyses in General Framing**

The present study employed two different timeframes (i.e., 1 yr and 10+ yrs) in the general condition to follow the method used by prior studies (e.g., Deng et al., 2022; Shrikanth & Szpunar, 2021). To understand how different timeframes may influence participants’ collective temporal thought, a post-hoc exploratory 2 (temporal domain: collective memory, collective future thought) × 2 (country: U.S., China) × 2 (age: younger adults, older adults) × 2 (timeframe: 1yr, 10+ yrs) mixed-factorial analysis of variance (ANOVA) with temporal domain and timeframe as within-participants variables were conducted for just the responses in the general condition. Given the reason for conducting this analysis was to investigate the effect of timeframe, only the effects involving timeframe are reported here. The ANOVA revealed that participants listed a lower proportion of positive events for the 1-yr timeframe (*M* = .55, *SD* = .37) than for the 10+ yrs timeframe (*M* = .63, *SD* = .34), *F*(1, 456) = 38.88, *p* < .001, 95% CI [.05, .10], *η^2^_p_* = .079. This result is not surprising considering that the latter timeframe covered a longer time span (Deng et al., 2022) and was less susceptible to the influence of COVID-19. In addition, there was a significant Timeframe × Country interaction, *F*(1, 456) = 12.90, *p* < .001, *η^2^_p_* = .028. The difference between the two timeframes was bigger for American participants (1 yr *M* = .33, *SD* = .27; 10+ yrs *M* = .44, *SD* = .28; 95% CI [.08, .15]) than for Chinese participants (1 yr *M* = .82, *SD* = .29; 10+ yrs *M* = .85, *SD* = .26; 95% CI [.001, .07]). In other words, American participants were more optimistic about the remote timeframe than about the near one, whereas Chinese participants did not prefer one timeframe over the other, albeit being overall more positive. Neither Timeframe × Temporal Domain interaction (*p* = .22) nor Timeframe × Temporal Domain × Country interaction (*p* = .40) was observed.

Consistent with prior research (Deng et al., 2022; Mert et al., 2022; Shrikanth & Szpunar, 2021), the current study included two different timeframes in the general condition to probe if participants may think differently about their nation’s near vs. remote past and near vs. remote future. Importantly, participants were more positive for the remote (i.e., 10+ yrs past and future) than near collective temporal thought (i.e., 1 yr past and future), which replicated Deng et al.’s (2022) finding. Moreover, the Timeframe × Country interaction demonstrated a significant difference between American participants’ near and remote collective temporal thought but only a marginal difference between Chinese ones. In other words, Americans were more positive about their remote collective temporal thought than about the near one, whereas Chinese were equally and highly positive about their near and remote collective temporal thought. Since there was no interaction between the timeframe and temporal domain, it is unclear if these timeframe differences happened mainly in collective memory or future thought. To better understand the underlying factors and directions of these timeframe differences, future research should include temporal distance and examine its relationship with various sociocultural constructs (e.g., self-construal, and media exposure; Deng et al., 2022).

***DSS Scores Distribution Across Countries***

The distribution of DSS scores by country is shown in the violin plot below.
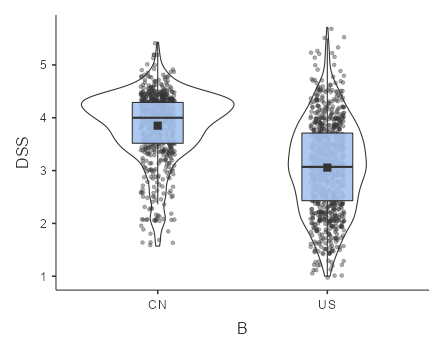

Supplement: Supplementary file 1 — Supplementary file1 (DOCX 57 KB) [file 13421_2024_1680_MOESM1_ESM.docx]
